# Entropy and Complexity Tools Across Scales in Neuroscience: A Review

**DOI:** 10.3390/e27020115

**Published:** 2025-01-24

**Authors:** Rodrigo Cofré, Alain Destexhe

**Affiliations:** Centre National de la Recherche Scientifique (CNRS), Institute of Neuroscience (NeuroPSI), Paris-Saclay University, 91400 Saclay, France; alain.destexhe@cnrs.fr

**Keywords:** entropy, complexity, neuroscience, maximum entropy principle, perturbational complexity index

## Abstract

Understanding the brain’s intricate dynamics across multiple scales—from cellular interactions to large-scale brain behavior—remains one of the most significant challenges in modern neuroscience. Two key concepts, entropy and complexity, have been increasingly employed by neuroscientists as powerful tools for characterizing the interplay between structure and function in the brain across scales. The flexibility of these two concepts enables researchers to explore quantitatively how the brain processes information, adapts to changing environments, and maintains a delicate balance between order and disorder. This review illustrates the main tools and ideas to study neural phenomena using these concepts. This review does not delve into the specific methods or analyses of each study. Instead, it aims to offer a broad overview of how these tools are applied within the neuroscientific community and how they are transforming our understanding of the brain. We focus on their applications across scales, discuss the strengths and limitations of different metrics, and examine their practical applications and theoretical significance.

## 1. Introduction

Our understanding of brain function has largely been shaped by the analysis of experimental data, from neuronal spikes and local field potentials (LFPs) to electrocorticography (ECoG), electroencephalography (EEG), and advanced imaging methods like voltage-sensitive dye imaging (VSDI) and functional magnetic resonance imaging (fMRI). In the absence of a unified theoretical framework in neuroscience, researchers have traditionally relied on statistical methods to analyze data [1]. As a result, neuroscientific discovery has often been data-driven, primarily focusing on characterizing variability or identifying correlations between recorded neural signals and the subject’s behavior, cognitive states, or sensory experiences. This approach has revealed exciting avenues for understanding the brain, even as we continue to search for unifying theories to tie everything together.

To move beyond traditional correlation analysis, entropy and complexity measures have emerged as powerful tools for quantifying the dynamic, nonlinear, and multiscale nature of brain activity across different levels of organization [2,3].

The concept of entropy, first introduced by Rudolf Clausius in the 19th century as a fundamental principle of thermodynamics [4], was transformed by Claude Shannon in 1948, who redefined it as a measure of information uncertainty in communication systems [5]. Shannon’s information theory extended entropy beyond its physical roots, providing a mathematical framework for quantifying unpredictability in *any* signal or probability distribution, laying the groundwork for its application in neuroscience. In this field, entropy has been used to analyze neural signals at different scales, for example, to quantify the unpredictability of spike trains [6,7], to quantify the variability in EEG rhythms [8,9], and to study the dynamic transitions between brain networks using fMRI [10,11], providing insights into the information-processing capabilities underlying sensory, motor, and cognitive functions.

Complexity encompasses a range of definitions across scientific fields, often linked to the amount of information or computational effort required to describe a system [12]. In neuroscience, complexity measures such as Lempel–Ziv complexity [13], neural complexity [14], and the Perturbational Complexity Index (PCI) [15] have been employed to capture the capacity of the brain for functional integration and segregation. Synergistic information further expands complexity measures in neuroscience, highlighting the importance of higher-order interdependencies that provide insights beyond simple pairwise interactions [16,17].

Despite the progress in applying entropy and complexity measures to study brain signals, there is still no comprehensive framework that integrates these concepts across different scales of brain organization. The current studies often focus on specific datasets or isolated levels of analysis, lacking a unified approach that connects single-neuron dynamics with macroscopic brain behavior. This fragmentation limits our ability to better understand how the brain processes information, adapts to changing conditions, and generates conscious experiences.

This review aims to synthesize the current research on the applications of entropy and complexity in neuroscience, highlighting how these measures can bridge gaps in our understanding of the brain’s multiscale organization. By reviewing the types of data used, as well as the variety of entropy and complexity indexes applied, we seek to provide a coherent picture of how these tools contribute to our current understanding of brain function. The insights gained from entropy and complexity measures offer the potential to unify findings across different experimental modalities and levels of analysis, thereby advancing the theoretical foundations of this field.

The review is organized as follows: we begin by summarizing the main types of recording techniques and neural signals used in neuroscience. We then introduce various entropy and complexity measures, detailing their mathematical formulations and applications in neuroscience. Finally, we discuss how these measures can be integrated to study brain function across multiple scales and propose future directions for research.

## 2. Types of Signals in Neuroscience

**Figure 1:**
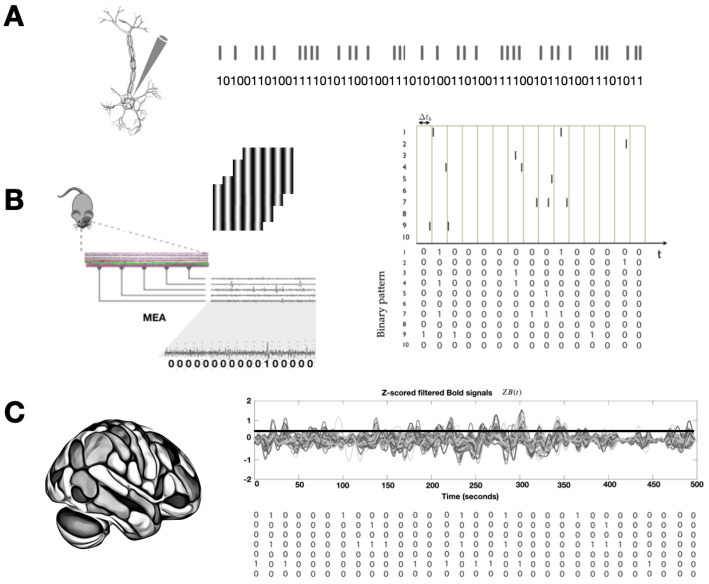
**Discrete signals.** (**A**) Single-cell spike recordings can be transformed into binary sequences of zeros and ones. (**B**) For simultaneous recordings from multiple neurons, such as those obtained via multi-electrode arrays (MEAs) in retinal ganglion cells responding to light stimuli, spike sorting is required to identify individual spikes. After selecting a binning time, a multidimensional binary signal is generated. (**C**) Continuous fMRI BOLD signals from a given parcellation can be discretized (e.g., assigning 1 to signals above 1 standard deviation and 0 otherwise) to create a multidimensional binary signal representing the whole brain.

Since entropy and complexity measures in neuroscience are typically derived from experimental data or computational models and simulations, it is important to understand the types of signals used to capture brain activity. These signals range from discrete to continuous, each offering distinct perspectives on neural dynamics across different spatial and temporal scales. Neuroscientists utilize a variety of recording modalities to probe brain function at multiple levels, from the activity of single neurons to the behavior of large-scale networks including the whole brain [18,19,20,21,22,23]. Below, we provide an overview of some of the key signals employed in the field (see Figure 1).

### 2.1. Discrete Signals

**Action Potentials (Spikes):** Neurons transmit information through action potentials, which are all-or-none events representing rapid depolarization followed by repolarization of the neuronal membrane potential. These events can be encoded as binary sequences, with a value of 1 indicating the occurrence of a spike and 0 indicating the absence of a spike, providing a discrete representation of neuronal firing [24].

**Spike Trains:** Temporal binary sequences that represent the occurrence of action potentials over time. Spike trains are usually recorded from individual neurons or groups of neurons, serving as a fundamental data type for analyzing neuronal activity patterns. They provide valuable insights into how single neurons and populations of neurons encode information [25].

**Raster Plots:** A graphical representation used to visualize spike trains across multiple neurons or experimental trials. In this type of plot, each row represents a neuron or trial, while each point indicates the timing of an action potential. This representation of spiking activity enables researchers to identify patterns of neuronal firing across different time intervals and experimental conditions [26].

**Symbolic Sequences of States:** A series of discrete symbols or states that represent a sequence of events or values over discrete time. Each symbol corresponds to a different state or category, and transitions between symbols capture changes in state over time. This approach is often employed to characterize transitions between discrete states of neurons or the entire brain and to simplify and analyze complex continuous data by converting them into a finite set of discrete states. In neuroscience, symbolic sequences of states are utilized to represent neuronal activity patterns [27] or brain states [10,28], which evolve over time.

### 2.2. Continuous Signals

**Figure 2:**
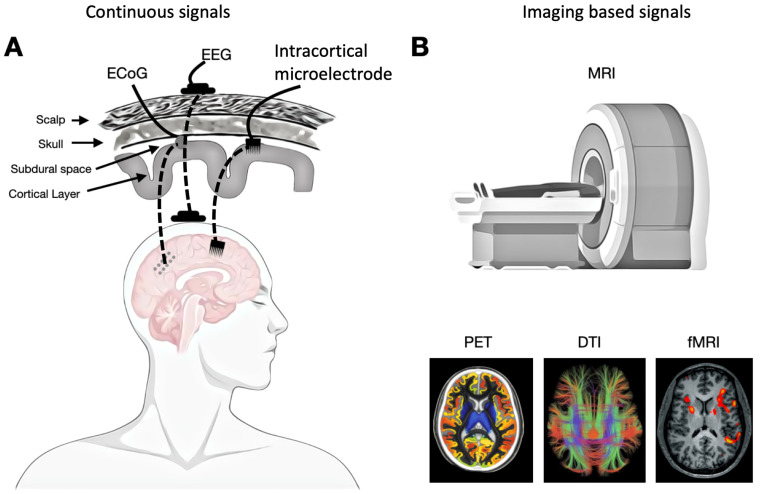
**Continuous and imaging-based signals.** (**A**) EEG signals are recorded using sensors attached to the scalp, detecting the brain’s electrical activity. Electrocorticography (ECoG), a type of intracranial EEG, involves electrodes placed directly on the exposed surface of the brain to capture electrical activity from the cerebral cortex. Implantable intracortical microelectrodes are surgically inserted into the cortex to record precise neural activity or stimulate specific groups of neurons. (**B**) Imaging-based signals, such as those from fMRI and DTI, are obtained using MRI machines, while PET scans reveal the metabolic and biochemical functions of tissues and organs.

Continuous signals provide analog data that capture neuronal or brain activity—either directly or indirectly—over time, providing insights into neural dynamics across various spatial and temporal scales. These signals reflect a wide range of neural processes, enabling researchers to investigate the continuous flow of information within the nervous system. By analyzing these signals through the lenses of entropy and complexity, we can gain a deeper understanding of the underlying mechanisms operating at multiple levels of organization (see Figure 2).

**Intracellular Recordings:** These recordings not only provide exceptional resolution in voltage (sub-millivolt), but also in time (sub-millisecond). By capturing the membrane potential (Vm), particularly subthreshold fluctuations that represent the synaptic inputs from thousands of synapses, intracellular recordings offer a rich continuous signal. This makes them an ideal source for applying computational methods to extract patterns and insights regarding the underlying neuronal and network activity. Recent results using entropy over these signals include the study of sensory processing by mechanoreceptors [29,30]. An insightful review on information theory and its use in neural coding at the single-neuron scale can be found in [31].

**Local Field Potentials (LFPs):** Continuous electrical signals that reflect the integrated synaptic activity of neuronal populations within a localized area surrounding the recording electrode [32]. LFPs capture slow oscillatory dynamics, including theta, delta, alpha, and gamma rhythms, which are essential for understanding the coordination of neural activity across regions. Recent developments show how to compute LFPs from integrate-and-fire network models, linking the micro- and mesoscopic scales [33,34].

**Intracranial Recordings (ECoG):** A highly invasive method involving electrodes placed directly on the cortical surface. ECoG captures continuous electrical signals representing the aggregate activity of neuronal populations with greater spatial and temporal resolutions compared to non-invasive methods (like EEG or MEG), enabling precise mapping of neural processes [35].

**Electroencephalography (EEG):** A non-invasive technique that measures the brain electrical activity via electrodes placed on the scalp. EEG recordings provide a continuous signal that represents the collective activity of large populations of neurons. This method is particularly valuable for analyzing brain rhythms, such as alpha, beta, theta, and gamma waves, which are associated with various cognitive and physiological states [36,37].

**Magnetoencephalography (MEG):** A non-invasive technique that detects the magnetic fields generated by neuronal currents. MEG provides continuous neural signals, similar to EEG, but offers superior spatial resolutions for localized brain regions, making it highly useful for studying the temporal and spatial dynamics of brain activity [38,39].

### 2.3. Imaging-Based Signals

Imaging-based signals refer to data acquired through various imaging techniques that visualize and measure biological processes associated with neuronal activity, brain function, metabolic processes, and structural connectivity. These signals provide insights into both the dynamic and static properties of the brain (see Figure 2).

**Calcium Imaging Signals:** Fluorescent imaging techniques that monitor calcium influx as an indirect measure of neuronal activity. Since calcium ions are involved in the activation of neurons, these signals provide continuous temporal data on neuronal dynamics in response to stimuli, enabling the visualization of activity across large populations of neurons [40,41].

**Voltage-Sensitive Dye Imaging (VSDI):** An optical technique for real-time monitoring of electrical activity across large populations of neurons. It relies on applying voltage-sensitive dyes to neural tissue, which fluoresce in response to changes in membrane potential. This technique provides continuous signals representing population activity across large areas of the brain [42].

**Functional Ultrasound (fUS):** A technique for imaging transient changes in blood volume across the entire brain, offering superior spatiotemporal resolution compared to other functional brain imaging methods [43]. Its ability to capture high-resolution, real-time data makes it particularly suitable for data-driven approaches in computational neuroscience.

**Blood-Oxygen-Level-Dependent (BOLD) Signal (fMRI):** A hemodynamic signal that reflects changes in blood oxygenation levels, serving as an indirect marker of neuronal activity. The BOLD signal is temporally coarse, capturing fluctuations on the order of seconds, and is widely used to investigate large-scale brain networks and functional connectivity during diverse cognitive states and sensory processes [44,45].

**Functional Near-Infrared Spectroscopy (fNIRS):** Is an optical brain monitoring technique that measures the oxygenation of a specific region of the brain to infer its activity. It uses infrared light to analyze the hemodynamic response, providing real-time information about brain activity [46]. A prominent application of fNIRS extends to both the diagnosis and treatment of psychiatric disorders [47].

**Diffusion Tensor Imaging (DTI):** An MRI technique that measures the diffusion of water molecules in tissues, particularly in the brain. It is based on the principle that water diffuses more freely along the direction of white matter fibers than perpendicular to them. By tracking this movement, DTI provides detailed maps of the brain’s white matter pathways, enabling the study of brain connectivity and structural integrity [48,49].

**Positron Emission Tomography (PET):** A nuclear imaging technique that measures metabolic processes in the body by detecting gamma rays emitted from a radioactive tracer. The tracer, typically a molecule like glucose labeled with a positron-emitting isotope (e.g., fluorine-18), is injected into the body and accumulates in areas with high metabolic activity, such as tumors or active brain regions. When the tracer decays, it emits positrons that collide with electrons, producing gamma rays that are detected by the PET scanner [50].

### 2.4. Computational Models and Simulations of the Brain

Computational models and simulations of the brain, spanning multiple spatial and temporal scales, serve as powerful generative frameworks for understanding and interpreting data in neuroscience. These models, often inspired by biophysical principles, aim to replicate neural dynamics by incorporating realistic structural and mechanistic features of neurons [51]. They are constructed at different levels of complexity, from the microscopic scale of individual neurons to the macroscopic scale of entire brain regions [51,52].

One prominent approach involves mean-field models, which provide a simplified yet effective representation of collective neuronal dynamics by averaging the behavior of large populations of neurons [53,54]. Such models capture the emergent properties of brain activity, including adaptation and responsibility to external stimuli [55], enabling the study of large-scale neural interactions without the computational burden of simulating individual neurons in detail. When coupled with the structural connectome—the detailed map of anatomical connections between brain regions—these mean-field models become highly informative tools for investigating the brain’s global dynamics, including network-level interactions and functional connectivity patterns in different brain states [56].

These models have the potential to simulate how disruptions in connectivity or neuronal dynamics might lead to pathological states, offering valuable mechanistic perspectives for understanding neurological disorders and developing therapeutic interventions from the perspective of entropy measures [57,58] or complexity [59,60].

## 3. Types of Entropy and Complexity Indexes in Neuroscience

Different types of entropy provide diverse insights into neural processes, from quantifying uncertainty to assessing regularity in neural data. In neuroscience, the primary focus is on entropy within the framework of information theory. Entropy, as a concept, spans various fields, each with its interpretations and applications.

### 3.1. Entropy

Without attempting to be exhaustive, we provide a taxonomy of the various types of entropy used in neuroscience research as follows:

**Shannon Entropy:** A measure of the uncertainty or unpredictability associated with a random variable [5]. It quantifies the information contained in a probability distribution and is widely used in information theory [61,62,63]. The more unpredictable or uncertain the outcome of a random variable is, the higher the entropy. The two extremes are the delta function with 0 entropy and the uniform distribution with entropy logN, where *N* is the number of possible outcomes of the random variable.

Given a discrete random variable *X* with possible outcomes $x_{1},x_{2},\ldots ,x_{n}$ and corresponding probabilities $P\left(X=x_{i}\right)=p_{i}$, the Shannon entropy H(X) is defined as

$$H\left(X\right)=-\sum_{i=1}^{n}p_{i}\log_{2}p_{i},$$

where H(X) is measured in bits when $\log_{2}$ is used and in nats when $\log_{e}$ (or the natural logarithm) is used.

If any $p_{i}=0$, the corresponding term in the summation is considered to be zero as $0\log_{2}0$ is defined to be 0 (using $\lim_{p\to 0}p\log p=0$).

Shannon entropy is particularly useful in neuroscience. Neural activity, such as action potentials (spikes) or brain rhythms (EEG), often carries information regarding sensory inputs, motor commands, and cognitive or consciousness states that can be quantified. Shannon entropy has found wide-ranging applications as an inference procedure in neuroscience, particularly through the lens of the Jaynes maximum entropy principle [62]. The core idea of the maximum entropy principle is to select the probability distribution that maximizes Shannon entropy, subject to known constraints—such as empirical averages or moments of observables—without making any additional assumptions about the system. Maximum entropy models have been used to infer the most likely distribution of neural firing patterns based on constraints such as average firing rates or pairwise correlations between neurons [64], triplets, and high-order correlations [65] and time-dependent correlations [66,67]. At the macroscopic level, the maximum entropy principle has been used to characterize resting-state human brain networks using fMRI data [68], to explore the energy landscape in brain network structure [69], and to study collective brain activity during wakefulness and anesthesia [70].

**Sample Entropy (SE):** A measure of complexity in time-series [71]. It assesses the probability that two sequences of data points that are similar over a given number of points remain similar when one more point is added.

Given a time series $\left\{x_{1},x_{2},\ldots ,x_{N}\right\}$, the SE is defined as

$$SE\left(m,r,N\right)=-\ln\left(\frac{A(m,r,N)}{B(m,r,N)}\right),$$

where *m* is the embedding dimension (length of the sequences to be compared), *r* is the tolerance for accepting matches, typically a percentage of the standard deviation of the time series, *N* is the length of the time series, *A* is the number of pairs of sequences of length m+1 that are within the tolerance *r*, and *B* is the number of pairs of sequences of length *m* that are within the tolerance *r*.

In neuroscience, sample entropy has proven to be a highly effective metric for quantifying the complexity of neural signals, including spikes, local field potentials (LFPs), and electroencephalography (EEG) recordings, particularly in the analysis of anesthesia depth [72,73]. Furthermore, it has been applied to MRI data to investigate neurodegenerative processes such as Alzheimer’s disease and the effects of aging [74] and to characterize functional complexity of fMRI human brain signals under propofol anesthesia [75].

**Multiscale Entropy (MSE):** A method to quantify the complexity of time-series data across multiple temporal scales. It extends the concept of entropy by examining how signal complexity changes when the data are viewed at different scales, which is important in understanding the dynamics of neural signals [76].

Given a time series $\left\{x_{1},x_{2},\ldots ,x_{N}\right\}$, for each scale factor τ, the coarse-grained time series $y^{\left(\tau \right)}$ is obtained by averaging the data points over non-overlapping windows of length τ

$$y_{j}^{\left(\tau \right)}=\frac{1}{\tau }\sum_{i=(j-1)\tau +1}^{j\tau }x_{i},\;j=1,2,\ldots ,\frac{N}{\tau },$$

where $\frac{N}{\tau }$ is the length of the new coarse-grained time series.

MSE is computed as follows: For each coarse-grained time series $y^{\left(\tau \right)}$, compute the SE, or another entropy measure, to quantify the regularity or complexity at that scale

$$S\left(\tau \right)=SE\left(y^{\left(\tau \right)},m,r\right),$$

The process is repeated for multiple scales $\tau =1,2,\ldots ,\tau_{\max}$, resulting in a set of entropy values S(τ) for different temporal scales.

Recently, multiscale entropy has been successfully employed to investigate retinal data in a mouse model of Alzheimer’s disease [77], as well as to analyze the complexity of brain activity in individuals with attention-deficit/hyperactivity disorder (ADHD) [78], and to study the human sleep cycle [79].

**Weighted Permutation Entropy (WPE):** Is a measure to characterize the complexity of nonlinear time series. It builds on Permutation Entropy (PE), which measures the randomness of ordinal patterns in a signal, by adding weights that account for the signal’s amplitude or energy. WPE is defined as [80],

$$H_{\mathrm{WPE}}\left(m,\tau \right)=-\sum_{i:\pi_{i}^{m,\tau }\in \pi }p_{w}\left(\pi_{i}^{m,\tau }\right)\ln p_{w}\left(\pi_{i}^{m,\tau }\right)$$

where *m* is the length of symbolic motifs in the time series, τ is a lag parameter indicating the number of time points to shift along the time series, $\pi_{i}^{m,\tau }$ represents the *i*-th symbolic motif out of the set of possible motifs, and $p_{w}\left(\pi_{i}^{m,\tau }\right)$ is the variance-weighted relative frequency of motif *i*. WPE has demonstrated broad applicability across neuroimaging modalities with varying spatial and temporal resolutions. For instance, it has been effectively utilized to analyze fMRI data, capturing spatiotemporal complexity in large-scale brain networks [81]. Similarly, WPE has proven valuable in studying EEG signals, where it provides insights into local neural dynamics and their temporal variability [82].

ϵ**-entropy:** A generalization of the Kolmogorov–Sinai entropy rate [83], which is defined for a finite scale ϵ and time delay τ by

$$\begin{array}{cc} h(\epsilon ,\tau ) & =\lim_{m\to \infty }h_{m}\left(\epsilon ,\tau \right)\\ \; & =\frac{1}{\tau }\lim_{m\to \infty }\frac{1}{m}H_{m}\left(\epsilon ,\tau \right), \end{array}$$

with $h_{m}\left(\epsilon ,\tau \right)=\frac{1}{\tau }\left(H_{m+1}\left(\epsilon ,\tau \right)-H_{m}\left(\epsilon ,\tau \right)\right)$. Here, $H_{m}\left(\epsilon ,\tau \right)$ represents the entropy calculated using a box partition of the phase space, where the box size is specified by ϵ, and the attractor is reconstructed with a time delay τ and embedding dimension *m*. For deterministic low-dimensional dynamics, this measure tends to plateau at specific scales. As such, it serves as a useful tool for characterizing large-scale dynamics while filtering out small-scale noise, and it can also aid in distinguishing chaotic behavior from stochastic noise under certain conditions [84]. In the context of neuroscience, ϵ-entropy has been used to study brain dynamics at multiple scales, and to show how one can reconcile the low-dimensional chaos of macroscopic variables (EEG) with the stochastic behavior of single neurons [85].

**Transfer Entropy (TE):** An information-theoretic measure used to quantify the directional transfer of information between two time series. It provides a way to capture the dynamic dependencies and causality between time-series signals, making it particularly useful for analyzing neural communication in the brain [86]. TE is often used as an alternative to correlation or mutual information as it specifically accounts for the influence of the past state of one variable on the future state of another variable.

Given two time-series $X_{t}$ and $Y_{t}$, the TE from *X* to *Y* (denoted $T_{X\to Y}$) is defined as

$$T_{X\to Y}=\sum_{y_{t+1},y_{t},x_{t}}p\left(y_{t+1},y_{t},x_{t}\right)\log\left(\frac{p(y_{t+1}|y_{t},x_{t})}{p(y_{t+1}|y_{t})}\right),$$

where $p(y_{t+1},y_{t},x_{t})$ is the joint probability distribution of the future state $y_{t+1}$, current state $y_{t}$, and the current state of $X_{t}$. $p(y_{t+1}|y_{t},x_{t})$ is the conditional probability of the future state of $Y_{t}$ given both its past state and the current state of $X_{t}$. $p(y_{t+1}|y_{t})$ is the conditional probability of the future state of $Y_{t}$ given only its own past state. TE quantifies the reduction in uncertainty regarding *Y*’s future by knowing the past of *X* beyond what is already known from *Y*’s past.

In neuroscience, TE can be used to map and analyze the directional information flow between different brain regions, helping to understand how neural networks process and transmit information, and to identify effective connectivity between brain regions, which is essential in understanding the neural mechanisms [87,88] and directed network inference [89].

**Entropy Production:** A quantity linked to temporal irreversibility in a system. In a system exhibiting temporal irreversibility, the forward trajectory is distinct from the reversed trajectory, leading to positive entropy production. This asymmetry indicates the inherent directionality of natural processes (arrow of time). The greater the entropy production, the more pronounced the temporal irreversibility of the process [90,91,92].

The entropy production denoted Φ can be defined in the context of information theory and statistical mechanics. For discrete Markov processes, it is often computed as follows:

$$\Phi =\frac{1}{2}\sum_{i,j}\left(p_{i}P_{ij}-p_{j}P_{ji}\right)\ln\left(\frac{p_{i}P_{ij}}{p_{j}P_{ji}}\right),$$

where $p_{i}$ and $p_{j}$ represent the invariant probability of the system being in states *i* and *j*, respectively, and $P_{ij}$, $P_{ji}$ the Markov transition rate from state *i* to state *j* and from state *j* to state *i*. The term $\left(p_{i}P_{ij}-p_{j}P_{ji}\right)$ represents the detailed balance difference, quantifying the net flow between states *i* and *j*.

In neuroscience, entropy production has been employed to investigate brain dynamics and to gain insights into how neural systems transition between different states under varying conditions and at different scales. In spiking neuronal networks, entropy production has been quantified using maximum-entropy Markov chains [93] and applied to characterize non-equilibrium steady states [94]. At the whole-brain level, entropy production has been used to characterize brain dynamics across different cognitive tasks [95] and to distinguish levels of consciousness in the human brain [96,97].

### 3.2. Complexity in Brain Dynamics

Complexity metrics, beyond entropies, help to capture emergent behavior. Here, we describe various complexity measures and their relevance in neuroscience.

**Lempel–Ziv Complexity (LZC):** A measure used to quantify the complexity or unpredictability of a sequence. It is based on the principles of the Lempel–Ziv compression algorithms, which are designed for lossless data compression. LZC evaluates how compressible a sequence is by counting the number of distinct substrings or phrases required to represent the sequence [98,99].

Given a sequence *S* of length *N*, Lempel–Ziv complexity $C_{LZ}\left(S\right)$ is computed by first decomposing the sequence *S* into a set of phrases or substrings such that each substring is either new or a repeat of an already observed substring. Then, the complexity is quantified by counting the number of distinct phrases or substrings needed to reconstruct the sequence.

Mathematically, if D(S) represents the number of distinct phrases required to represent the sequence *S*, then the Lempel–Ziv complexity is provided by

$$C_{LZ}\left(S\right)=\frac{D(S)}{N}.$$

This formula provides a normalized measure of complexity, reflecting the proportion of distinct phrases relative to the total length of the sequence. Sequences with low LZC values are more repetitive and compressible. For example, sequences with many repeating patterns or regular structures will exhibit low complexity. Sequences with high LZC values are less compressible and exhibit a higher degree of unpredictability.

LZC is particularly useful for analyzing various types of neuroscientific data, providing insights into their underlying complexity and structure. Among the many applications in neuroscience, it has been used as an alternative entropy estimator for binned spike trains [100], for fMRI data of propofol anesthesia of spontaneous brain activity in rats [101], in EEG recordings to discriminate sleep and wakefulness [102], and in MEG data to analyze the effects of external stimulation on psychedelic state neurodynamics [103].

**Algorithmic complexity:** Algorithmic complexity, or Kolmogorov complexity, quantifies the complexity of a data object (e.g., a string, image, or dataset) as the length of the shortest computer program capable of reproducing it. Unlike Shannon entropy, which describes the statistical properties of a distribution, algorithmic complexity captures the intrinsic structure and compressibility of individual objects [104]. In neuroscience, algorithmic information theory offers a framework for understanding how the brain enables structured experience and self-awareness. Consciousness is proposed to emerge from the brain’s role as an information processor, balancing complexity to optimize adaptability and efficient information integration.

This theoretical lens aligns naturally with multiscale neural dynamics, where algorithmic complexity provides insight into information integration across spatial and temporal scales. Methods for studying conscious states include assessing spontaneous activity, perturbation-based experiments, and behavioral metrics. Together, these approaches leverage algorithmic complexity to bridge the gap between neural processes and structured experience, complementing other information-theoretic frameworks [105].

**Figure 3:**
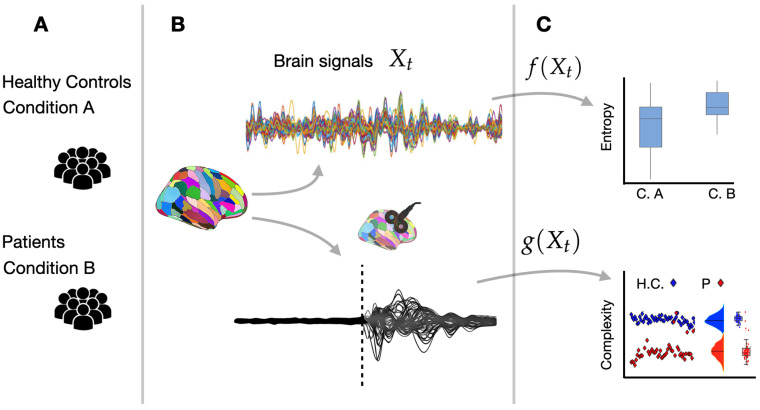
**From brain signals to entropy and complexity.** (**A**) Two groups are typically analyzed: either a population of healthy controls versus patients with a particular pathology, or a single population under varying conditions, such as placebo versus drug treatment. (**B**) Brain activity data—such as EEG, MEG, or fMRI—are collected from these groups under different experimental conditions, for example, before and after external stimulation. (**C**) Entropy and complexity measures are then computed as functions of the acquired data (denoted here as functions *f* and *g*). These measures enable the comparison of brain signal characteristics, revealing potential differences between groups (e.g., Condition A (CA) versus Condition B (CB) or healthy controls (HCs) versus patients (Ps)).

**Perturbational Complexity Index (PCI):** A measure used to quantify the complexity of brain responses to external perturbations, such as Transcranial Magnetic Stimulation (TMS), and it is often applied in the study of consciousness [15,106,107], as well as in models [59]. The index reflects the diversity and complexity of spatiotemporal patterns that result from brain activity. Low PCI indicates a stereotypical simple brain response, often observed in unconscious states such as general anesthesia, coma, or deep sleep. In contrast, high PCI indicates a complex, varied brain response, generally observed in conscious and awake states (see Figure 3 for illustration).

### 3.3. Steps to Compute PCI

1. **Perturbation:** Apply external stimulation to a brain region.
2. **Recording:** Measure the resulting brain activity.
3. **Spatiotemporal Analysis:** Analyze the recorded data to extract binary spatiotemporal patterns.
4. **Compression:** Apply a compression algorithm (such as Lempel–Ziv complexity) to the spatiotemporal patterns.
5. **Normalization:** Normalize the compressibility score to obtain the PCI.

Let *B* represent the binary spatiotemporal pattern derived from the recordings. The Perturbational Complexity Index (PCI) can be defined as

$$\mathrm{PCI}=\frac{\left(C(B)\right)}{\max\;\left(C\right)},$$

where C(B) is the Lempel–Ziv complexity (or another compression measure) of the spatiotemporal pattern *B*, and max(C) is a normalization factor representing the maximum possible complexity of the system’s response, ensuring PCI values range between 0 and 1.

One of the most impactful clinical applications of PCI is in the evaluation of patients with disorders of consciousness (DOCs). Patients in a coma typically exhibit low PCI values, reflecting the brain’s inability to generate complex, integrated responses. PCI has been used to distinguish between Unresponsive Wakefulness Syndrome (UWS) and minimally conscious state (MCS). Patients with UWS usually have low PCI values, while minimally conscious patients often show slightly higher complexity, indicating some preserved capacity for integrated brain function. PCI can help to detect locked-in patients (who are fully conscious but unable to move) as locked-in patients typically have PCI values similar to those of conscious individuals.

**Neural Complexity (Tononi’s Complexity):** Introduced by Giulio Tononi and colleagues [14], it is a measure that quantifies the balance between integration and differentiation in a neural system, but, unlike PCI, this measure does not require any external perturbation of the brain. Neural complexity reflects how well the components of a system can interact while maintaining their functional specialization [108].

Consider a bipartition of the system *X* into a *j*-th subset $X_{j}^{k}$, composed of *k* components, and its complement $X-X_{j}^{k}$. The mutual information (MI) between $X_{j}^{k}$ and $X-X_{j}^{k}$ is

$$MI\left(X_{j}^{k};X-X_{j}^{k}\right)=H\left(X_{j}^{k}\right)+H\left(X-X_{j}^{k}\right)-H\left(X\right),$$

where $H(X_{j}^{k})$ and $H(X-X_{j}^{k})$ are the entropies of $X_{j}^{k}$ and $X-X_{j}^{k}$ considered independently, and H(X) is the entropy of the system considered as a whole. Mutual information MI is 0 if $X_{j}^{k}$ and $X-X_{j}^{k}$ are statistically independent and MI>0 otherwise.

The neural complexity NC(X) is defined as the average of mutual information over all bipartitions of the system

$$NC\left(X\right)=\sum_{k=1}^{n/2}\langle MI\left(X_{j}^{k};X-X_{j}^{k}\right)\rangle .$$

Neural complexity is high when a system shows rich interactions between its parts and diverse specialized activities within those parts. This measure is particularly relevant to studies of consciousness and brain organization, where both high integration (coordinated activity across the brain) and high differentiation (specialized processing in different regions) are important features [109].

**High-order Interdependencies:** A measure that capture complex multi-variable relationships that extend beyond pairwise interactions [17]. Traditional graph representations, where edges represent pairwise interactions between nodes, are insufficient to fully describe high-order interactions. These more intricate dependencies, involving three or more variables at once, are better modeled by hypergraphs or simplicial complexes, which are mathematical tools capable of representing multi-variable interactions [110].

Let $X_{1},X_{2},\ldots ,X_{n}$ be a set of random variables with joint probability distribution $P(X_{1},X_{2},\ldots ,X_{n})$. The Total Correlation (TC) [111] is provided by

$$TC\left(X_{1},X_{2},\ldots ,X_{n}\right)=\sum_{i=1}^{n}H\left(X_{i}\right)-H\left(X_{1},X_{2},\ldots ,X_{n}\right)$$

where $H(X_{i})$ is the Shannon entropy of the *i*-th random variable, and $H(X_{1},X_{2},\ldots ,X_{n})$ is the joint entropy of all the variables. Alternatively, it can be written as the Kullback–Leibler divergence $D_{KL}$ between the joint distribution and the product of the marginal distributions

$$TC\left(X_{1},X_{2},\ldots ,X_{n}\right)=D_{KL}\left(P\left(X_{1},X_{2},\ldots ,X_{n}\right)\|P\left(X_{1}\right)P\left(X_{2}\right)\ldots P\left(X_{n}\right)\right)$$

The Dual Total Correlation (DTC) [112] is provided by

$$DTC\left(X_{1},X_{2},\ldots ,X_{n}\right)=H\left(X_{1},X_{2},\ldots ,X_{n}\right)-\sum_{i=1}^{n}H\left(X_{i}|X_{\setminus i}\right),$$

where $H(X_{i}|X_{\setminus i})$ is the conditional entropy of $X_{i}$ given all other variables $X_{\setminus i}$, defined as

$$H\left(X_{i}|X_{\setminus i}\right)=H\left(X_{1},X_{2},\ldots ,X_{n}\right)-H\left(X_{\setminus i}\right).$$

The O-Information [113] (denoted Ω) is defined as

$$\Omega \left(X_{1},X_{2},\ldots ,X_{n}\right)=\left(n-2\right)H\left(X_{1},X_{2},\ldots ,X_{n}\right)-\sum_{i=1}^{n}H\left(X_{i}|X_{\setminus i}\right)$$

If Ω>0, the system is *redundancy-dominated*; otherwise, if Ω<0, the system is *synergy-dominated*. Recent applications of these concepts in the context of neuroscience include a study of high-order interdependencies in the aging brain [114], neurodegeneration [115], cognitive state and behavior in the macaque cerebral cortex [116], and a study of the synergistic workspace for human consciousness [117].

### 3.4. Key Differences Between Entropy and Complexity in Neuroscience

Entropy primarily measures uncertainty, disorder, or randomness in neural activity. It provides insights into the predictability of brain states and is useful for understanding transitions between different cognitive states, such as consciousness, attention, or sleep. Complexity instead focuses on the organization and interactions of neural components. It reflects how well the brain’s neural networks are integrated and how flexible or adaptable the brain is in processing information. See Table 1, for comparisons of entropy and complexity measures in neuroscience.

## 4. Challenges and Limitations

While entropy and complexity approaches offer powerful insights into brain function, they also present several challenges and limitations in neuroscience research. These issues are methodological and related to data quality and quantity, interpretation, and the generalization of findings.

### 4.1. Data Quality and Arbitrary Preprocessing

Neuronal recordings are often noisy and prone to artifacts (e.g., head movement, electrical interference, environmental disturbances, etc.). Because entropy and complexity measures are directly computed from data, they are sensitive to signal quality. Artifacts or noise in data can lead to spurious entropy or complexity values. In particular, entropy measures may incorrectly increase in the presence of noise, leading to an overestimation of disorder or unpredictability not intrinsically reflecting a property of the neuronal populations. A way to deal with this issue is to filter the data to eliminate the noise, but this introduces another issue related to arbitrary choices regarding filtering. Signal filtering and detrending can significantly influence the outcomes of entropy and complexity, sometimes leading to non-replicable results [118].

Effective preprocessing steps, such as artifact removal, normalization, filtering, and signal alignment, are essential for extracting meaningful information. However, improper handling of these steps can introduce biases, distort the underlying neural signals, and lead to spurious conclusions when computing entropy and complexity measures. For example, in electrophysiological experiments, spike sorting and baseline correction can significantly impact the results of data analysis [119]; in neuroimaging, choices related to motion correction or spatial smoothing can drastically affect brain activity patterns [120,121].

### 4.2. Choosing Appropriate Parameters

Some entropy and complexity measures require specific tuning of parameters. Sample entropy and multiscale entropy are highly dependent on user-defined parameters, and choosing these parameters can be non-trivial (embedding dimensions, time-lags, or scale factors). Entropy computed from symbolic sequences depends on the choice in thresholds for each symbol, and the number of symbols is usually arbitrary [122]. Arbitrary or improper selection of parameters can lead to misleading results, making cross-study comparisons difficult. There is often no clear or universally accepted method for selecting these parameters, and optimal values can vary depending on the nature of the data (e.g., different recording modalities, recording sites, and recording lengths).

### 4.3. Interpretation of Results

While entropy and complexity measures provide quantitative insights, their biological interpretation remains challenging. High entropy may suggest randomness or flexibility; understanding what this means in terms of brain function or behavior requires careful examination [123]. The interpretation of increased or decreased entropy/complexity depends heavily on the scale and neurobiological context. For example, increased entropy might reflect pathological dysfunction (e.g., in epilepsy [124]) or healthy adaptive variability (e.g., during cognitive tasks [125] or psychedelic states [126,127]). Without a clear framework, it is difficult to assign biological meaning to these changes in entropy.

### 4.4. Experimental and Computational Implementation

Entropy and complexity measures, especially when applied at multiple scales, can be computationally expensive and time-consuming, requiring elaborate experimental setups and sophisticated offline computations, often necessitating parallel computing or advanced optimization techniques [15,64]. This drawback makes these measures difficult to apply in the clinical context, where rapid and accurate results are needed. Another example is calculating higher-order interdependencies, which presents a significant challenge due to the combinatorial explosion that occurs as the number of variables increases. This exponential growth in the number of possible interactions makes it increasingly difficult to evaluate every potential interdependency, requiring substantial computational resources or alternative computations that bypass the complete exploration of interactions.

### 4.5. Non-Stationarity of Brain Signals

Neural data are often non-stationary, meaning that the statistical properties of the signals (e.g., mean and variance) change over time. This presents a significant challenge for entropy and complexity approaches, which often assume stationarity, making it difficult to determine whether changes in these metrics reflect genuine neural variability or artifacts of non-stationarity [128,129]. While techniques such as windowing or adaptive measures exist to address non-stationarity, they add another layer of computation and introduce potential biases in the results.

### 4.6. Comparability Across Studies and Modalities

Different brain imaging modalities (e.g., EEG, fMRI, and MEG) operate at distinct temporal and spatial scales, and the interpretation of entropy and complexity measures may differ depending on the recording modalities. Comparing results across different studies or modalities is challenging. For example, entropy or complexity measures calculated from EEG (which has high temporal but low spatial resolution) may not be directly comparable to those derived from fMRI (which has high spatial but low temporal resolution). Another issue mentioned before that impacts comparability across studies and modalities is the absence of standardized approaches to calculate and report entropy and complexity measures. Even subtle differences in preprocessing or parameter choices can result in divergent conclusions, complicating meta-analyses or replication efforts [120].

### 4.7. Pathological vs. Healthy Brain States

Although entropy and complexity measures are useful for distinguishing between healthy and pathological states, such as in epilepsy [124] or neurodegenerative diseases [130], the distinction is not always rigorously defined. Reduced entropy is often associated with neurodegenerative disorders, but this relationship may not be linear or consistent across all brain regions or stages of disease. The challenge is understanding when entropy changes are pathological and when they reflect adaptive processes. In some pathological states, increased entropy may represent compensatory neural mechanisms rather than dysfunction. Entropy increases during psychedelic experiences in healthy and pathological populations, reflecting heightened neural complexity and variability, which add further complexity to the interpretation [131].

### 4.8. Statistical Challenges and False Positives

Some entropy measures may not perform well with small datasets or short time series. Most complexity measures require long and continuous recordings to provide stable results, limiting their use in some experimental paradigms with limited time resolutions like fMRI [132]. When applying entropy measures across multiple brain regions, time points, or scales, the risk of false positives increases due to multiple comparisons. Proper statistical corrections (e.g., Bonferroni or FDR) are necessary but can sometimes obscure meaningful findings [133,134].

## 5. Discussion

In recent years, significant advancements have been achieved in neuroimaging techniques and electrophysiological recordings, enhancing both their quantity and quality across diverse spatial and temporal scales [135]. Despite this important progress, neuroscience remains without a unified theoretical framework, leaving the field in a “data-rich, theory-poor” state [136]. This disparity between abundant data and the lack of comprehensive theories underscores the need for data-driven approaches to extract meaningful insights. Such approaches enable the discovery of patterns and relationships that might otherwise remain hidden.

In this review, we have highlighted examples of the practical applications of entropy and complexity metrics in neuroscience. The clinical implications of these approaches are extensive and promising. Recent studies demonstrate that the entropy of brain signals can differentiate states of consciousness in neurological and psychiatric disorders [28,137,138,139,140]. These findings suggest that entropy-based metrics could serve as sensitive biomarkers for detecting consciousness, supporting the hypothesis that neural complexity may be a fundamental aspect of human consciousness [75,109]. One of the most widely validated complexity measures with clinical utility is the Perturbational Complexity Index (PCI) [15,107], which has emerged as an objective marker of consciousness (see [59] for models). The PCI provides clinicians with a valuable tool for assessing awareness levels in non-communicative patients, especially when behavioral assessments are unreliable or restricted. Entropy has also been used to model how psychedelics induce altered consciousness. The Entropic Brain Hypothesis, proposed by Robin Carhart-Harris and colleagues [126,127], suggests that different states of consciousness correspond to varying levels of entropy, or disorder, in brain activity. Psychedelics like psilocybin and LSD are believed to increase brain entropy. This entropy shift may underlie the profound subjective experiences reported during psychedelic states, such as expanded consciousness and a sense of interconnectedness, offering insights into how psychedelics affect brain function at a fundamental level. This high-entropy brain activity may enable altered perceptions, enhanced creativity, and novel cognitive insights. Therapeutically, such high-entropy states may help individuals to break free from rigid thought patterns, offering alternatives for treating conditions like treatment-resistant depression or PTSD [141].

Additionally, we provide the reader with a critical perspective on using complexity metrics. While entropy and complexity metrics are valuable tools for exploring brain dynamics, their application requires careful consideration as their effectiveness depends on the methodological rigor of analytical workflows. These metrics, while flexible, are sensitive to factors such as signal noise, data quality, and preprocessing choices, which can significantly impact the results. As such, careful experimental design and rigorous statistical controls are essential to ensure that entropy and complexity indexes reflect genuine aspects of brain activity rather than artifacts. Furthermore, standardizing methodologies and creating transparent, reproducible data-processing pipelines are critical as neuroscience increasingly embraces large-scale datasets and sophisticated computational methods. Such rigor is fundamental for facilitating meaningful comparisons across studies and building a cumulative understanding of neural complexity.

A critical consideration in the study of entropy and neural signal variability is the distinction between true neural variability and measurement noise, as discussed in Section 4. This distinction is particularly important because noise can introduce biases into entropy computations, potentially obscuring key insights into brain function. However, an increasing body of evidence suggests that neural variability is not merely noise but a crucial dimension of functional dynamics of the brain [22]. As argued in [142], neural variability represents a key, yet undervalued, factor in understanding brain-behavior relationships. They emphasize that such variability reflects the brain’s capacity to adapt flexibly to internal and external demands, making it a fundamental property for understanding behavior at both inter- and intra-individual levels. Similarly, in [81], they provide a complementary perspective by demonstrating how complexity dynamics underlie the brain’s functional network organization. Their study identifies spontaneous “complexity drops” in fMRI signals, which represent transient episodes of neural regularity that shape connectivity strength, network topology, and structure-function relationships. Notably, their analytic framework addresses challenges related to the non-stationarity of brain signals, a limitation discussed in Section 4. These works underscore the dual importance of neural variability and signal complexity as complementary perspectives for understanding the dynamic architecture of brain function and its role in cognition and behavior.

The future of entropy and complexity metrics in neuroscience is highly promising. With advances in recording technologies and increasingly accessible computational resources, these tools are set to take a central role in decoding the brain’s intricate dynamics. Their integration with the abundant experimental data (“data rich”) and sophisticated algorithms is emerging as a transformative force in scientific discovery, as evidenced by recent Nobel Prizes [143,144].

This novel scenario may open new avenues for clinical applications, including personalized diagnostics and treatments for neurological and psychiatric disorders driven by better experimental datasets and data generated by sophisticated brain simulations [145,146]. Additionally, the development of more sophisticated models for understanding brain function and consciousness, informed by these metrics, may lead to groundbreaking discoveries that bridge the gap between experimental data and theoretical developments. We hope that, with continued progress, these tools will not only deepen our understanding of brain function but also revolutionize the way we diagnose, monitor, and treat brain-related conditions.

## Figures and Tables

**Table 1:** Comparison of Entropy and Complexity in Neuroscience.

Aspect	Entropy	Complexity
**Definition**	Entropy in neuroscience refers to the *degree of unpredictability, disorder, or uncertainty* within neural activity, often quantifying the randomness in brain states.	Complexity refers to the *organizational richness and interactive dynamics* among neural components, reflecting the brain’s ability to integrate information and adapt to varying cognitive demands.
**Focus**	Measures the *randomness and unpredictability* of neural firing patterns or brain activity.	Focuses on the *integration, interdependence, and organizational structure* of neural networks, reflecting higher-order cognitive processing.
**Extreme values**	Maximal for totally random systems (lack of statistical structure).	Maximal for systems with a mix of order and disorder (intricate statistical structure).
**Units**	Measured in *bits* (information theory), or *nats*.	Often unitless.
**Role in Brain Function**	Provides insights into the *predictability* of brain states, helping to characterize cognitive transitions between states like attention, sleep, and consciousness.	Characterizes *organizational complexity and flexibility* of the brain, emphasizing how well neural systems can adapt and process complex, dynamic information.
**Relation to Brain states**	High entropy: A chaotic or disorganized state of neural activity, such as during epileptic seizures or psychedelic states. Low entropy: A highly predictable state, such as general anesthesia or deep sleep.	High complexity: Adaptive and flexible brain states, such as creative thinking and cognitive flexibility. Low complexity: Diminished cognitive function or highly constrained cognitive states.
**Relation to Disorders**	High entropy has been associated with disordered brain states (e.g., during epileptic seizures), while low entropy is often seen in conditions with total or partial loss of consciousness (e.g., general anesthesia, sleep, disorders of consciousness).	Low complexity is frequently observed in *neurodegenerative diseases*, where the integration of brain networks is impaired, leading to cognitive decline also in patients with disorders of consciousness. High complexity reflects *healthy brain function and adaptability*.

## Data Availability

Data is contained within the article.

## Abbreviations

The following abbreviations are used in this manuscript:

LFP	Local field potential
ECoG	Electrocorticography
EEG	Electroencephalography
fMRI	Functional magnetic resonance imaging
fNIRS	Functional Near-Infrared Spectroscopy
fUS	Functional ultrasound
PCI	Perturbational Complexity Index
MSE	Multiscale Entropy
LZC	Lempel–Ziv Complexity
KL	Kullback–Leibler
MEP	Maximum Entropy Principle
MEA	Multi-Electrode Array
SE	Sample Entropy
TE	Transfer Entropy
DTC	Dual Total Correlation
TC	Total Correlation
UWS	Unresponsive Wakefulness Syndrome
MCS	Minimally Conscious State

**Symbol List**
H(X)	entropy of the random variable *X*;
$p_{i}$	probability of outcome $x_{i}$;
$T_{X\to Y}$	Transfer Entropy from *X* to *Y*;
Φ	entropy production;
NC(X)	neural complexity;
$C_{LZ}\left(S\right)$	Lempel–Ziv complexity of the sequence *S*;
Ω	O-Information.
